# Why do Chinese women experience gamophobia? Psychoanalytic theory assisted discourses analysis

**DOI:** 10.3389/fpsyg.2024.1357795

**Published:** 2024-04-03

**Authors:** Joanna Nian Chang

**Affiliations:** Shanghai Jiao Tong University, Shanghai, China

**Keywords:** gamophobia, Chinese young women, social media, psychoanalytic theory, discourses analysis

## Abstract

Social media is currently abuzz with discussions about the topic of women’s gamophobia in China. Nevertheless, there is few research investigating gamophobia from a psychological perspective. This study utilizes content analysis and sentiment analysis to examine and analyze 879 individuals’ texts about gamophobia on Little Red Book and uses psychoanalytic theory, which is centered on comprehending and interpreting the psychological processes of the human mind, to investigate the elements that contribute to women’s gamophobia, aiming to address this knowledge gap. This investigation revealed that gamophobia might exert physical, psychological, and several other effects on individuals. This study employs a psychoanalytic framework and concludes that the rise of independent consciousness, many unhappy marriages in their environment, anxiety about dealing with unfamiliar family relationships, pursuit of personal and professional development, original family issues, changing perceptions of aging care, the media effect, the concept of parenthood, and criteria for choosing a life partner are the nine primary factors contributing to women’s gamophobia. To address the societal issues and outcomes resulting from a fear of marriage, it is advisable for those who experience this phobia to examine their negative defensive mechanisms and prioritize rational thinking in their mindset. Moreover, the Government should establish a social atmosphere that ensures women are neither influenced nor constrained by the media. Furthermore, promote holistic family education to bolster the self-awareness and prospective family comprehension of young individuals. Finally, government departments should also offer promotion and supporting measures to help assuage women’s concerns about marriages.

## 1 Introduction

Gamophobia refers to the fear or worry that arises while considering marriage, establishing a long-term relationship, or making a commitment to another person (Obeid et al., 2020). The marriage rate is a significant metric for evaluating society’s progress since it exhibits a substantial correlation with factors such as population growth and crime reduction (Rader, 2010; Liu et al., 2023). China is currently undergoing a persistent decline in its marriage rate. In 2022, China experienced a significant decline in its marriage rate, hitting its lowest level in 37 years. According to the Ministry of Civil Affairs of the PRC, all together, there were only 6.833 million couples who were officially married in 2022. Regarding geographical disparities, economically affluent regions exhibit a comparatively reduced marriage rate. Beijing contributed 1.34% to the overall national marriage rate in 2022, while Shanghai accounted for 1.05% and Tianjin represented a mere 1%. As a reaction to the persistent decline in marriage rates, the Chinese government has implemented a range of policies in an effort to alleviate this tendency. Currently, there are 32 provinces and cities, including Sichuan, Hebei, and Shanghai, that have been explicitly designated as “experimental zones for reforming marriage customs.” The intention of these zones is to tackle outdated marriage practices, such as lavish wedding banquets and exorbitant dowries, in order to assist young individuals in developing a more appropriate perspective on marriage. On special events like “520” (homophonic I love you in Chinese), “Tanabata” (Chinese Valentine’s Day), and “Valentine’s Day” that occur on weekends, civil affairs agencies in numerous locations extend their operating hours to facilitate the registration of marriages for newlywed couples. On May 18, 2023, the State Council authorized the expansion of the “cross-province marriage registration” pilot project to meet the increasing needs of mainland citizens who had moved. “Cross-province marriage registration” was put into effect in 21 provinces.

Although the government is actively working to update and modernize the legislation pertaining to the marriage law, concerns around marriage have become a contentious subject of public perception. The topic of “gamophobia” has garnered significant attention on social media, amassing a combined total of over 100 million views. An example of such platform is Little Red Book, a Chinese social media platform that bears similarities to Instagram and Pinterest. Users of Little Red Book can share their own experiences and interact with other users by posting short videos and images. However, unlike Instagram and Pinterest, Little Red Book allows its users to opt for complete anonymity by using their account. During registration, Little Red Book system assigns each user a number, such as 123,456, by default. If desired, users have the option to change this number to a username of their choice. Indeed, several users also choose to preserve their anonymity and evade identification from those who have firsthand acquaintance with on Little Red Book. This implies that users will have a greater sense of independence while sharing material on the social media, as they will not have to be concerned about being evaluated by others, such as coworkers and family members. Anonymity promotes individuals to authentically showcase their true lives instead of participating in a fabricated act. Therefore, after a span of just 10 years, beginning in 2013, Little Red Book has risen to become the second most popular social media application in China. The latest data shows that the Monthly Active Users (MAU) of Little Red Book has exceeded 260 million. The core user demographic comprises mostly young girls, accounting for around 80 percent of the total user base, generally falling between the age range of 18 to 35. The prevalent motif of a “gamophobia” on Little Red Book partially mirrors the psychological condition, perplexity, or distress experienced by many young women while facing the reality of marriage.

In a study conducted by iResearch in 2020, it was found that just 14% of people indicated a positive inclination toward marriage and a sense of eagerness for getting married. Conversely, 36% of respondents reported apprehension toward marriage, specifically due to certain unfavorable societal perceptions linked with it. Based on the 2022–2023 Report on Chinese Men and Women’s Attitudes toward Marriage, more than 36% of unmarried adults encounter marriage-related anxiety (iResearch, 2023). The highest proportion of marriage anxiety is observed among persons born after 1990 in this category. In addition, a study by Youth Culture Observe IP on the opinions of Chinese youth regarding marriage revealed that 77% of them believe they are unfit for marriage, while 54.2% are indifferent to the institution of marriage and neither actively oppose nor support it. Furthermore, gamophobia is not limited to China. Based on the U.S. Census Bureau (2020), the marriage rate in the United States has reached its lowest level in 150 years, which was 9.8 per 1,000 individuals in 1990. Nevertheless, the rate has declined to 6 by 2021 (Statista Research Department, 2023). South Korea exhibits a notable degree of unease toward marriage, as seen in the fact that the total count of newlywed couples in 2022 amounts to slightly over one million. There was a decrease of over 30 percent in comparison to the year 2015. In addition, 46% of couples who entered into their first marriage did not have any offspring (Yonhap News Agency of Korea, 2022). These findings indicate that the general societal issue of fear toward marriage has become prevalent on a global level.

The increasing prevalence of fear of marriage poses a huge challenge to China’s social norms and social development. Nationally, China’s marriage rate drops to 4.8 percent in 2022, below the world average of 5.4 percent. In terms of the age structure of the population, China’s age at first marriage has been significantly delayed. In 1990, it was 22.87 years old, of which 23.59 years were for men and 22.15 years for women. The average age of first marriage was 28.67 years old in 2020, of which 29.83 years old for men and 27.95 years old for women. There has been a 6-year increase in the average age at which people get married over a period of 30 years (Marriage Industry Insights, 2023). In Chinese traditional society, those who remain unmarried after reaching the age of 30 often encounter verbal censure from their older family members. Now it is normal. Notably, the average age at which individuals experience their first romantic love has consistently decreased throughout time. Specifically, the average age decreased from 21.2 years old in the post-1970s generation to 17.4 years old in the post-2000s generation (Century Jiayuan, 2023). There appears to be a divergence in the comprehension of affection across different generations. The present-day Chinese youth’s perception of marriage has diverged from the goals and anticipations of traditional Chinese families and society. Which variables have influenced this deviation? What are the consequences of people and society experiencing dread or aversion toward marriage? In order to alleviate the anxiety experienced by young individuals when it comes to marriage, it is essential to perform a thorough scientific investigation of both the phenomenon of gamophobia and the specific demographic group affected. This evaluation will hold considerable academic importance and practical value in improving comprehension and reducing the anxiety surrounding marriage among young individuals.

Curis and Susman (1994) categorized the reasons for this fear into ten distinct groups: fear of loss sense of self, identity, and emotional engulfment; fear of loss of control or being controlled; fear of reenactment or duplication of the aversive parental marriage; fear of financial losses and complications related to inheritance; worry about underperforming in the marriage and the potential consequences of divorce; unease about aging and physical decline in the presence of their partners; fear of their own dependence and vulnerability; apprehension about betrayal, rejection, and abandonment; reluctance to take on the responsibilities of family and marriage; and a lack of self-assurance and belief in one’s ability to achieve a happy marriage independently. According to Kefalas et al. (2011), women with higher education place a greater emphasis on their careers than on getting married and starting a family. Cohabitation has emerged as a viable option for many young individuals as an alternative to marriage (Willoughby and Carroll, 2012). Due to the absence of official acknowledgment of living together, both individuals do not need to worry about domestic chores. This enables them to dedicate more time and focus to their personal interests and enjoy the freedom of being single. The 2012 National Survey of Family Growth (NSFG) data showed a notable rise in the percentage of cohabitation, increasing from 3% in 1982 to 11% from 2006 to 2010 (Copen et al., 2012). Furthermore, cohabitation does not act as a precursor to marriage. Many young people hold the view that marriage is not a mandatory component of life and that choosing not to marry can be a conscious decision (Dai and Chilson, 2022). Empirical data suggests that less than 25% of young individuals of both genders who cohabit ultimately enter into matrimony (Schoen et al., 2007). In addition, as society progresses, the pace of work and daily life continues to escalate. Modern everyday life is characterized by work pressure, information overload, emotional strain, financial pressure, and other comparable concerns. The notable upsurge in daily life stress has led to a considerable rise in the mental health burden that many people are currently bearing. According to Lancet, in 2021, the global prevalence of clinical depression will surpass 246 million individuals. Moreover, the prevalence of female individuals affected is approximately twice that of males (COVID-19 Mental Disorders Collaborators, 2021). Psychopathology suggests that clinical depression leads to reduced motivation and lower commitment to personal relationships, resulting in a feeling of separation from marital partnerships (Obeid et al., 2020).

In China, the attitude toward marriage is closely connected to the practical aspects of one’s life. The high occurrence of pragmatic difficulties, such as extravagant housing expenses, employment barriers, educational impediments for children, pension obligations, healthcare worries, and restricted social interaction, has resulted in an increased feeling of anxiety among young adults. Intense societal pressure discourages young adults from getting married (Lin, 2019). Moreover, the correlation between technology and capital in the media sector has led to the commercialization of women’s concerns, transforming them into a show driven by financial gain (Cao and Dai, 2022). In order to garner greater attention in the media, many content creators willingly perpetuate binary gender boundaries. It reinforces the division between genders in reality and undermines women’s desires for authentic marriage (Wu, 2020). The study on marriage among Generation Z adolescents demonstrates a significant negative correlation between their amount of online involvement and their desire to get married. Furthermore, it was observed that women belonging to Generation Z exhibit a greater degree of apprehension toward marriage when compared to men (Lan et al., 2023).

In the Chinese cultural setting, marriage entails a woman’s obligation to join the man’s family, which unavoidably involves navigating the dynamics of his familial relationships. Additionally, the woman assumes the responsibility and potential challenges associated with parenting. Hence, comprehending women’s gamophobia is an essential requirement for resolving the issue. Nevertheless, there is a scarcity of research on women’s discourse regarding fear of marriage, with the majority of studies examining conversations between males and females collectively. Furthermore, the fear of marriage is a social and psychological issue. However, upon reviewing the existing literature, it is evident that there is a dearth of research on the micro-psychological aspects of this fear. Most studies primarily concentrate on analyzing the effects at the macro-social level. Hence, in contrast to prior research, this paper’s potential addition is evident in two aspects: (1) This study aims to examine the factors contributing to women’s apprehension about marriage, offering a female viewpoint and empirical evidence to analyze the issue of gamophobia and address the declining marriage rate. (2) Elucidating and scrutinizing the issue of women’s fear toward marriage through the lens of a social psychology study, thus enhancing the investigation into this matter.

## 2 Materials and methods

The widespread adoption of communication technology has deeply infiltrated the daily lives of the general population through various forms of media. Media has evolved beyond being a neutral entity and now has the power to influence society (Yu and Geng, 2021). Social media is essential in the media ecosystem as it removes geographical constraints, allowing people from various social groups to access information and express their emotions. This phenomenon alters the nature of public space, makes it more intimate, and erases the clear boundary between the individual and society. Meyrowitz’s investigation in *No sense of place* reveals that the electronic realm has blurred the boundaries between the physical world and the virtual world. There has been a discernible rise in the prevalence of emotional problems that were formerly regarded as personal in recent years. This encompasses the proactive dissemination of insignificant, day-to-day, personal adverse sentiments and anecdotes on social media platforms. An instance of this can be seen in the discussion surrounding the concept of “gamophobia.” Yin and Meng (2018) found that social media users are more likely to pay closer attention to fearful and unpleasant emotions. The presence of anxious and negative public attitudes is not limited just to the domain of social media public opinion but also affects the perception of marriage among young individuals in reality. Moreover, individuals communicate their opinions on the Internet, bypassing the influence of interviewers and having more freedom in articulating their emotions. Thus, this work employs a research methodology that integrates both quantitative and qualitative approaches to investigate the discourse of people who have gamophobia. This study seeks to clarify the primary factors and outcomes of Chinese women’s reluctance toward marriage from a social psychology standpoint. In addition, it proposes strategies to alleviate women’s concerns about marriage, promoting personal welfare and social unity.

### 2.1 Data collecting

This study utilizes Little Red Book as the research platform and employs Python to extract the textual content related to four prevalent topics of gamophobia: “gamophobia” (110 million views), “do not want to get married” (54.244 million views), “everyday gamophobia” (17.304 million views), and “fear of marriage in the post-90s generation” (13.735 million views). The aim is to examine the variables that impact the apprehension toward marriage among females. The choice of Little Red Book as the data source platform is predicated on two factors: (1) The user base of Little Red Book is significant and actively involved. The site has a total of 260 million users who are active on a monthly basis, with 69 million individuals who regularly share material. (2) The user demographics of Little Red Book are remarkable, since the majority (80.06%) consists of women, and a significant percentage (70%) comprises those born in the 1990s. This is in line with the research focus of this paper, which centers on single women.

Additionally, this study did not utilize a traditional search engine. Conversely, the study sample consisted of statements that were popular and garnered over 10 likes. The selection of the screening sample was based on the number of likes, since a higher number of likes signifies a higher level of recognition for the content of the text (Yao et al., 2021). The Python extraction process retrieves many data elements, such as keywords, title, tweet details, likes, favorites, comments, shares, hashtags, posting address, posting time, genre, author, author’s profile, gender, and author’s address. By implementing Python de-weighting, excluding likes, conducting gender screening, and manually eliminating tweets unrelated to the issue, we have acquired a grand total of 879 texts that are both highly liked and of exceptional quality. These texts have received a total of 782,800 likes, 167,000 favorites, 140,200 comments, and 71,800 shares. The data covers the time period from November 27, 2020, to August 27, 2023.

### 2.2 Methodology

#### 2.2.1 Psychoanalytic theory

Psychoanalytic theory, a prominent field of psychology established by Jewish Austrian neurologist Freud Sigmund in the early 20th century, Psychoanalytic theory forms the basis of modern social psychology, focusing primarily on the examination of human psychological activities. Therefore, this study utilizes psychoanalytic theory to investigate the phenomenon of gamophobia among contemporary Chinese women.

#### 2.2.2 Content analysis

This study primarily employs the content analysis approach to encode and analyze 879 texts. Subsequently, it utilizes a combination of psychoanalysis theory to develop research conclusions, ultimately pinpointing the crucial components of Chinese young women’s gamophobia. The process is separated into three distinct steps: firstly, a classification system is established; secondly, statistical analysis is conducted on the features of the content; and finally, suggestions are made based on the conclusions drawn from the study.

#### 2.2.3 Sentiment analysis

Sentiment analysis has become an essential technique for understanding the emotional components of data. Sentiment analysis is based on analyzing the frequency of words. In order to improve the understanding of text, the first step is to use the ROSTCM6 software to carry out word segmentation. This involves applying a specific vocabulary filter to exclude unimportant phrases such as “of” and “and.” Manually incorporate synonyms for self-love, such as love yourself, love of self, and self-love. Gooseeker, uses the given material to identify positive and negative emotions. Gooseeker as a tool of analysis, employs its established syntactic conventions and computational algorithms to calculate emotional values for words, as well as evaluate emotional inclinations. Upon identifying negative emotional words, the texts were manually classified according to their semantic meaning. Data is provided to understand the impact of gamophobia in young females.

## 3 Factors and repercussions of women’s gamophobia

After analyzing the content of the 879 text materials and considering the purpose of the study, it has been ascertained that Chinese women’s gamophobia can be attributed to the following nine factors:

### 3.1 Rise of independent consciousness

ID 17: My parents still think it’s abnormal not to get married, but now I enjoy the life. Despite the occasional despondency over a broken light bulb high above, a new piece of furniture I cannot put together, or a large bucket of water I bought that I struggled to lift, But it is free. Have time to think, have enough energy to study, and cook a big meal to treat myself. It is a life experience; try to make money, do good exercise, maintain a good state of mind, have a healthy body, and really feel happy already. (emphasizes the value of personal autonomy)

ID 100: I really do not like the idea of getting married and having kids because it’s a very heavy thing to do. It means that you have to integrate into another family and share your private space, and especially after having children, it seems that you will definitely sacrifice yourself. Although the above concessions and sacrifices may also be accompanied by joy and happiness, often they are actually big gambles. Because marriage is really a neutral word that can elevate your life like never before, but it also has the potential to plunge your life into unimaginable predicaments. (fear of losing self-boundaries)

ID 127: 26 years old. My life has no mother-in-law and daughter-in-law relationship, no endless cooking, I can work without burden, I can see music festivals, go to the sea, go crazy, and enjoy without worry. Such a life, I do not want to get married. I hate to get married; I love to be free and easy on myself. (enjoying freedom)

ID 197: I have spent my life first becoming myself and then playing the important role of a full-time daughter! I do not want to get married! (loves myself)

### 3.2 Many unhappy marriages in their environment

ID 104: My friend and her husband fight every day over money to get a divorce, and I am very resistant to getting married. (financial problems)

ID 208: At home, I heard a child crying loudly in the hallway, just to go out to see several neighbors in the coaxing of the child. The child seems to be two or three years old, not wearing shoes. Then, when the child’s mother went out to pick up a delivery, the child’s father also left after mom went out, leaving the child at home by himself. The child opened the door to come out (see too many accidents; really glad that the child did not go out the window). Neighbors said the child cried for almost an hour; his parents’ phones could not be reached; after a while, the child’s mother rushed back; she was completely unaware of the situation, but also by the neighbors a scolding. (men’s lack of parental responsibility)

ID 526: a couple. Woman: This dress is still wet, right? It was washed last night. The man did not say anything. Woman: Today will be cold, so you should wear a jacket. The man still did not say. Inside me, it is the man of thirty or forty years old, not a three- or four-year-old baby. Really just suffocating, more fear of marriage day. (family trivia)

ID 527: A sister said that she has to take her children to school in the morning, then come to work. When off work, she needs to buy food, cook, do the dishes, mop the floor, and then do the laundry. Then her husband came home to eat dinner and play with the phone and computer. I am afraid of marriage. (Men lack family responsibilities).

### 3.3 Anxiety about dealing with unfamiliar family dynamics

ID 423: The possibility of experiencing conflicts and strains with my mother-in-law and daughter-in-law after being married strongly discourages me from getting married. (concern about the interactions and relationship with a mother-in-law)

ID 524: Love and marriage are separate. When it comes to marriage and one’s family, it is necessary to make a conscious effort to actively participate in their matters, which can be rather tiring. (Prefer maintaining a specific amount of separation).

### 3.4 Original family issues

ID 471: I went to my grandparents’ house for dinner, and as soon as I walked in the door, I saw my dad playing cards with my grandpa, my younger uncle playing his phone on the couch, and my grandma working in the kitchen all by herself. My mom and I sat down for 2 min and then went to help my grandma. (paternal machismo)

ID 493: I’m 29 years old, and I finally bought my own house, which is not very big, but I’m glad I’m on my own. I grew up with an incomplete family, and my mom’s unfortunate marriage has made me fearful of marriage. Sometimes I envy my friends who are happily married. But I have no confidence. (parental failed marriage)

ID 556: As a result of my parents’ pressure, I am currently adamantly opposed to matchmaking and apprehensive about marriage. (parents pushing for marriage)

### 3.5 Pursuit of personal and professional development

ID 112: loss of job and possible job advancement opportunities, or competitiveness in choosing a new job. (loss of competitiveness in the workplace)

ID 539: If I give birth, I will lose my job, and I will become a disgruntled woman at home, and I do not have much hope for marriage when I think about it. (fear of losing job)

### 3.6 Changing perceptions of aging care

ID 134: Simply configure an insurance policy for myself to increase protection, but also let mom and dad have medical insurance. I can use the “insurance” to cover my own old age. (commercial insurance for protection)

ID 301: I’ll be 24 in a few months. From now on, I will take care of my body and make money to live in a good nursing agency so that I can maintain a good quality of life when I grow old. (earn money for own retirement)

### 3.7 Media effect

ID 520: I just read that a man yelled at his wife, who just had a cesarean. The next article says that the man yelled at his wife, who was seven weeks pregnant. It is possible to get married just to find a free nanny who can do the childcare, laundry, and cooking, plus earn money. No, modern women should seek equality in inequality and realize their value in all aspects. (negative marriage messages on social media)

### 3.8 Concepts of parenthood

ID 499: Whenever I see children on bicycles or playing with a ball in the park, I cannot help staying away from them. I really do not like children at all. Do not try to persuade me (I do not like children).

ID 536: I cannot be a good parent myself. (parenting worries)

ID 824: I was born in 1994. I think DINK is really good. But most guys cannot accept not having kids. I’m afraid to get married because I do not want to have kids. (I do not want to have kids).

ID 919: I’m afraid of pregnancy. Especially the belly cannot be retracted; a belly full of stretch marks, horrible contractions, and bloated milk. (fertility worries)

### 3.9 Criteria for choosing a life partner

ID 77: I want sincerity and favoritism, but this is full of hypocritical boys. (insincere)

ID 125: He only comforts me: “do not think too much.” Daily communication is about eating. No emotional intelligence, and it does not provide any emotional value. But, my family always tells me that marriage is realistic. (unable to provide emotional value)

**Figure 1:**
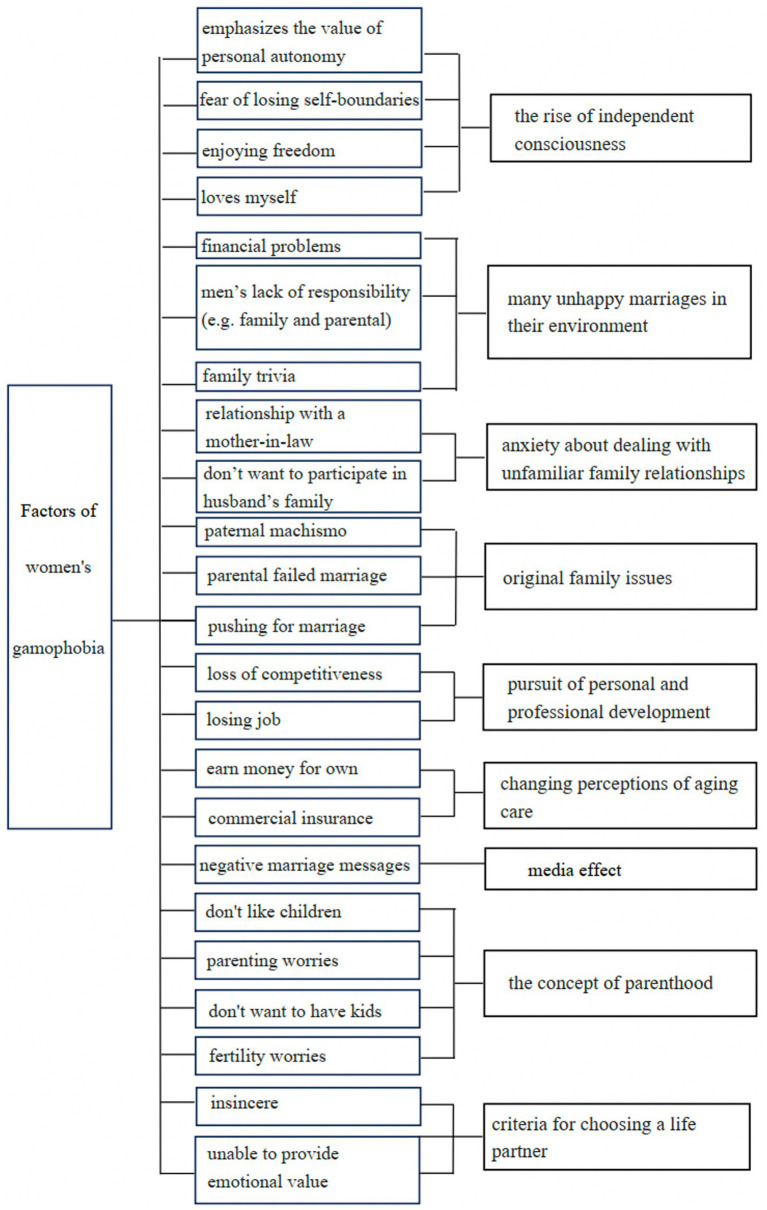
Factors of women’s gamophobia.

This article analyzes and assesses 879 case texts and concludes that the reasons behind Chinese women’s uneasiness toward marriage can be categorized into nine distinct groups (see Figure 1): the rise of independence consciousness, many unhappy marriages in their environment, the anxiety about dealing with unfamiliar family relationships, original family issues, the pursuit of personal and professional development, the changing perception of aging care, media effect, the concept of parenthood, and the criteria for choosing a life partner. Gamophobia has detrimental effects on many women, resulting in individual psychological distress and pressures. Even women who have planned for marriage also have concerns, leading to a significant amount of psychological stress.

ID 10: I was looking forward to going home for New Year’s Eve, but when I think of the blind date, it’s still good to think that I might not be able to go back for New Year’s Eve this year. A little afraid.

ID 37: My family introduced me to a blind date. I do not want to go out to see people.

ID 328: The closer I get to marriage, the more I do not want to get married. Am I the only one like this?

ID 337: My mom told me to see a psychiatrist. There are no more unmarried people like my age, and my parents are ashamed of me. They say now I’m a leftover woman. Do I really need to find someone to marry now? I do not even dare to breathe at home.

ID 430: I think it’s good to be alone! Now I do not even want to talk to boys.

These detrimental effects do not occur in isolation. Among the 879 replies that were analyzed, psychological stress, worries, numbness, insecurity, panic, rebelliousness, and terror, was reported a total of 575 times. The occurrence of social distress, characterized by aversion toward social outings and blind dates, as well as a diminished level of social interests, was explicitly referenced a total of 66 times. Furthermore, there were 109 instances where individuals indicated subsequent consequences such as aversion to marriage, apprehension toward marriage, the dissolution of relationships, and episodes of crying. Clearly, the fear or anxiety over getting married may have a big negative impact on a person’s mental health. A noteworthy aspect is that Chinese society recognizes gamophobia as a moral infraction, and the sense of fearing marriage is regarded as a negative emotion (Zhu, 2008).

However, the discourse around apprehensions about marriage encompasses more than just unfavorable implications. It also includes feminist concepts like self-assurance, autonomy, and self-love among Chinese young women.

ID 28: I’m really happy to be 28! Although my pockets are empty and my money is spent on renting a house and drinking and eating, A lot of people my age choose to get married and have babies; they have their happiness. I have my own happiness.

ID 73: Just respect yourself in the moment, even if you cannot prepare for it for the rest of your life.

ID 109: I have worked for the college entrance exams, felt the joy of going to college, experienced the dilemma of entering the workplace, and gloated over the small achievements of my career. I have left my hometown and had the honor of enjoying the beautiful scenery of my country, and I have also set foot on overseas land to struggle in a new environment. I know that there are all kinds of people in the world, colorful lives, and countless possibilities. Standing on the shoulders of my parents, I have worked hard and struggled a little bit to become the person I am today. I know how hard it was for my parents, and I know how hard it was for me. I know more clearly what I will lose than what I may gain from marriage.

ID 144: 27 years old is really much better than 18 years old; feel the freedom like never before; go wherever you want; only need to consider your own happiness or not happiness to make decisions; live freedom! I do not want to get married!

**Figure 2:**
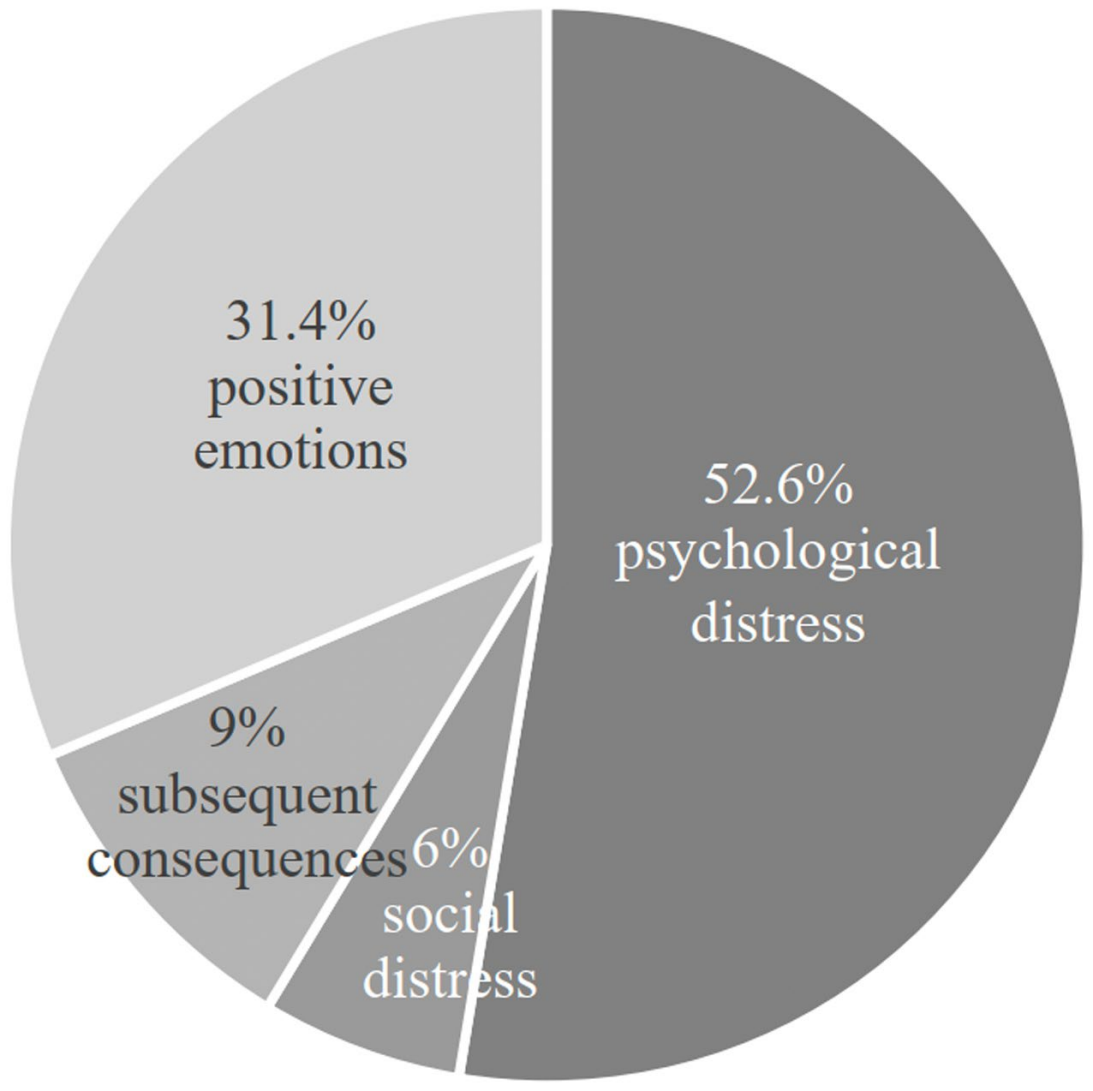
Repercussions of women’s gamophobia.

Although the positive emotions did not surpass the bad repercussions, certain factors such as self-assurance, self-esteem, autonomy, self-congruence, authenticity, and joy were mentioned a total of 343 times. Nevertheless, it is apparent that many women view their hesitation toward marriage not as a negative emotion but rather as a purposeful choice to control their own lives. Figure 2 presents a statistical chart that depicts the impact of the fear of marriage.

## 4 Psychoanalytic analysis of the factors and repercussions of women’s gamophobia

Psychoanalysis, developed by Freud, is a complete framework that elucidates the formation of personality and provides a psychotherapy approach. It includes ideas such as the theory of the unconscious mind, personality, sexual desires, the theory of dream interpretation, and the defense mechanism of repression. According to Freud, the formation of personality is rooted in libido and defensive instincts. The psychologist’s objective is to understand this inherent urge. Irrespective of whether an individual’s innate abilities are operating at an unconscious, preconscious, or conscious level, they generate energy and guide behavior. As Freud pointed out, personality can be divided into three separate components: id, ego, and superego. Id is the main source of all cognitive energy and represents a portion of the unconscious mind. Id’s functions in terms of the pleasure principle and strives for instant enjoyment. As a result, id might be described as basic, inherent, or essential. The role of the id is more prominent during one’s childhood. The ego, a constituent of consciousness, acts as a suppressor and controller of “id” and protects from potential harm. As humans grow older, they gain the wisdom to avoid acting on impulse. The superego is the pinnacle of the personality, embodying the ultimate standard and upholding ethical principles. It offers direction to the ego and enforces limitations on the id. As individuals develop, they gradually adhere to moral, educational, and legal standards. Freud proposed that defensive mechanisms consist of many techniques such as repression, negation, projection, regression, isolation, displacement, rationalization, compensation, sublimation, humor, and reaction formation. The defensive mechanism primarily serves as a safeguarding function of the ego. Individuals often face a dilemma as they navigate between tangible reality and their ambitions, leading to internal struggles and inconsistencies. The ego resolves these conflicts and contradictions to accommodate the superego while also satisfying the id’s wishes. The objective of this approach is to diminish anxiety, mitigate distress, and surmount psychological obstacles. This study utilizes Freud’s psychoanalytic theory to elucidate the factors that contribute to Chinese women’s gamophobia, as well as the resulting outcomes, as illustrated in Table 1.

**Table 1:** Psychoanalytic theory examines Chinese women’s gamophobia.

Texts		Theoretical analysis
Factors contributing to women’s gamophobia	Criteria for choosing a life partner, media effect	Id
	The rise of independent consciousness	Ego
	Many unhappy marriages in their environment	Rationalization
	Anxiety about dealing with unfamiliar family relationships	Imbalance in the relationship between id, ego, and superego
	Original family issues	Repression
	Changing perceptions of aging care, pursuit of personal and professional development	Displacement
	Fertility worries and parenting worries	Imbalance in the relationship between id, ego, and superego
Repercussions	Anxiety, numb, insecure, etc.	Regression
	Social distress, stay at home	Isolation
	Confidence, freedom, self love, etc	Reaction formation

### 4.1 Id

According to Freud, human beings have two primary instincts: “Eros” (libe instinct) and “Thanators” (defense instinct). “Eros” pertains to the innate propensity of human beings to partake in actions that yield gratification and evade those that induce distress. “Thanators” relates to the inherent need for self-preservation and safeguarding. In the era of new media, diverse sorts of multimedia, including images, video, animations, and others, are employed to convey negative news items concerning marriage. This enables users to engage in a more immersive encounter with “symbolic reality.” These symbolic realities have a cultivation effect on female users, reinforcing a gloomy picture of marriage. Multiple researchers have confirmed the cultivation effect of media in their studies, demonstrating that the accumulation of negative news broadcasts about marriage in the media leads women to disenchant marriages (Zhang and Xiao, 2008; Dai and Lv, 2021). When women are unable to find a suitable partner, this, along with their failure to find a compatible marriage, activates their inherent need to safeguard themselves from harm. A joke that circulated online, “Abstaining from marriage and not having children will result in eternal youth” and “Abstaining from marriage and not having children will protect one from harm,” may seem humorous, but they also function as a way for women to instinctively set a fear-driven limit on marriage.

### 4.2 Ego and reaction formation: resistance to patriarchy

Autotheory is rooted in feminist and queer theory (Fournier, 2021). The process stems from personal direct experiences, disregards prejudiced assumptions, and its examination cannot be separated from specific texts or activities (Sweet, 2021). Therefore, in the field of autotheory, many scholars use the conflicting effects of tools such as mirrors and selfies as evidence for the existence of the self (Gu, 2022). The competing influence emerges due to the discrepancy between the self-expressed through the tools and the genuine self, in accordance with the concept of ego in psychoanalytic theory. This concept aims to regulate the conflict between the id and the superego. This research is an analysis of how individuals who have concerns about marriage utilize autotheory on the social media platform Little Red Book (performative writing and social media interactions), and it reveals a keen awareness of the existence of patriarchy.

According to data from China’s seventh population census, the male population in China comprises 51.24%, while the female population comprises 48.76%. Although the overall female population is smaller than the male population, Since 2009, the number of women enrolling in master’s degree programs in China has surpassed that of men. The total enrollment of master’s degree programs in 2019 was 447,000, with female students comprising 55.1% of this figure (China Education Online, 2021). the progress of women’s education, challenging the traditional Chinese concept of male supremacy and female subjugation in marital unions as well. Upon inspection of the case text, it becomes evident that conventional factors such as family background and financial stability are no longer the primary determinants for women when selecting a partner in the present era. Women in the contemporary era prioritize emotional value, sincerity, commitment to agreements, self-worth, autonomy, and self-focus. The frequent use of phrases like “freedom” and “self” in the texts implies that women aspire to achieve emotional and marital satisfaction by cultivating their individual spirits. However, a sizable portion of men in China continue to adhere to traditional heterosexual norms and the machismo mentality, which makes them uncomfortable with the idea of marriage as feminism advocates. The divergence in viewpoints between males and females regarding marriage is a notable element that contributes to women’s unease toward marriage.

### 4.3 Rationalizations to explain dissatisfaction within a marriage

By creating justifications that appear logical, people use rationalization as a defense mechanism to protect themselves from setbacks. Additionally, they minimize behaviors and reasons that deviate from societal norms in order to free themselves. Marriage and family have always held significant value in traditional Chinese households and society, reflecting their goals and expectations. Expressing fear of marriage deviates from the traditional Chinese cultural emphasis on this institution, which prioritizes adherence to these principles. Therefore, the fear or unease regarding marriage is considered a violation of society and moral norms (Zhu, 2008). In order to alleviate their anxiety about not being married, many women try to hide their fear by emphasizing the rational reasoning that “even if those in their social circle do get married, most of them are unlikely to experience true happiness.” Therefore, some women may not have an innate fear of marriage but instead have concerns about not finding a compatible mate or being overwhelmed by the significant financial obligations that come with marriage. As a result, individuals link their problems to a fear of marriage and take defensive actions to protect themselves. According to a well-known saying, people often embrace a Confucian philosophy when they are happy and a Taoist philosophy when they are unhappy. Rationalization, on the other hand, is a philosophical methodology that entails adjusting to the trials and conditions of existence.

### 4.4 “Regression” and “isolation” as coping mechanisms for anxiety

Regression refers to the phenomenon when an individual, faced with irritation, stress, or fear, returns to a former stage of conduct that they had previously acquired during their earlier life. Regression, in contemporary psychoanalytic theory, refers to the reemergence of actions and experiences that occurred during the early childhood period, specifically between the ages of 0 and 6. Marriage apprehension frequently emerges as individuals reach the age at which they are anticipated to enter into matrimony. Parents’ demands that their children find a compatible partner and their refusal to accept anyone who does not live up to their expectations are the main causes of anxiety. Consequently, individuals may revert to a state like that of a kid, displaying heightened sensitivity, a tendency to cry, and a desire to seek emotional relief from their intense fear. Certain individuals choose to retreat, much like an ostrich burying its head in the sand, in order to evade the confrontation of conflict and rage. They isolate themselves as a means to mitigate the anguish they experience. As the wedding date approaches, those individuals who possess a phobia of marriage feel a sense of fear, like facing a powerful opponent. Nevertheless, they are unsure about how to tackle this matter and consequently resort to becoming emotionally unstable with their partners or possibly ending the relationship entirely.

### 4.5 A repression-upbringing environment

Freud defines repression as the unconscious process of suppressing conflicting or unsuitable emotions and upsetting occurrences from the conscious mind into the preconscious realm. Although the conscious mind may not be cognizant of it, repression endures as an emotion in the preconscious. Based on the case texts, it is evident that certain individuals with a fear of marriage were raised in households where the father displayed male chauvinistic behavior and the mother exhibited traits of patience, quarrelsomeness, and indifference. A dysfunctional family may instill in a child the conviction that marriage is an unpleasant and terrible struggle. Furthermore, this negative perspective on marriage continues to exist even as one ages, simply shifting to the preconscious level of the mind. As children develop, the unsuccessful marriages of their parents unintentionally influence their viewpoints on love and marriage. As a result, when these young individuals enter into relationships with their own partners, they may naturally experience feelings of uncertainty, doubt, and even a reduced sense of their own value and unease toward the interactions between genders. Their choice to refrain from marriage or evade long-term marital obligations is motivated by their “repression.”

### 4.6 Anxiety impedes a vocation and self-care

Displacement, also known as alleviation, is the use of a symbolic object or behavior to counteract something unpleasant and maintain a positive mental state. The case text reveals that individuals who fear marriage often redirect and project their anxiety and discomfort onto work-related difficulties. Furthermore, the notion of “rear sons for help in old age” has transitioned to embracing one’s own aging process. The concerns about getting married are balanced by the potential risk of not having a support system in old age, which can be reduced by using commercial pension insurance and other preventive measures. The “China Women’s Pension and Risk Management White Paper” [Fudan Development Institute (Manulife-Sinochem), 2020] reveals a consistent upward trend in the proportion of women’s yearly earnings allocated toward commercial pension insurance. The current rate stands at 5.89% and is expected to increase to 8.87% in the future.

### 4.7 Imbalance in the relationship between id, ego, and superego

Based on the case text, the study identifies an imbalance between “Id,” “Ego,” and “Superego,” notably a dominance of the “Id” over the “Superego.” This perspective on marital fear argues that marriage is a type of disadvantage and bondage. When individuals engage in the legal agreement of marriage, they must accept the possibility of becoming parents, dealing with new family relationships, handling household duties, and taking on the responsibility of raising children. The laws and responsibilities linked to these marriages generate apprehension, self-questioning, and a feeling of incongruity with one’s own aspirations in individuals who are afraid of making a commitment. Hence, they hold the belief that remaining single or cohabiting is more conducive to comfort than entering into marriage. This discrepancy becomes evident when they choose to renounce marriage.

## 5 Conclusion and suggestions

In recent times, feminism has begun to gain momentum and evolve in China. The expansion can be linked to factors such as the enhancement of women’s economic status, expanded availability of ideological education, and the impact of media communication. Consequently, the standing of women’s voices has experienced an unprecedented enhancement. Little Red Book, a prominent social media platform in China, consistently showcases the authentic viewpoints of young women to the public. Hence, this study holds practical and theoretical importance as it investigates the current perspectives on marriage among young women through the observation and analysis of their apprehension toward marriage expressed on Little Red Book. It also aims to provide tailored interventions and services based on the findings.

Firstly, it is crucial to first understand their defense mechanisms and then provide suitable solutions. Individuals should refrain from explicitly attributing the defects in other people’s lives to their life partner, despite the unpleasant sentiments expressed by their parents and friends. Individuals can openly express their concerns and fears to their partner and work together to cultivate a deep emotional connection rooted in trust. By embracing this strategy, you can shield yourself from trivial hardships in your environment and the media and effectively strengthen yourself against the harmful effects originating from your family heritage.

Moreover, according to the feminist viewpoint presented by Japanese sociologist Chizuko Ueno, feminism essentially involves unconstrained independence and the pursuit of personal satisfaction, regardless of one’s wishes or constraints (Ueno and Reiko, 2023). Women have the option to choose marriage willingly, and their choice to abstain from it should not lead to prejudice or ill-treatment toward individuals who have misgivings about getting married. Hence, it is imperative for the government to take proactive and deliberate steps to guide the general population in progressively overcoming the limitations imposed by outdated gender norms through media. This includes dispelling the notion that women’s higher education levels are primarily responsible for changes in marriage patterns. It is vital to give women the proper respect and allow them the independence to marry and have children. According to the research samples, Chinese women who experience apprehension toward marriage have realized that marriage phobia is not intrinsically detrimental. Furthermore, they have acknowledged that an individual’s marital status has no bearing on their overall optimistic perspective on life. By actively striving to improve their own growth and development while implementing appropriate risk management and protective measures, individuals can also achieve happiness.

Additionally, education is the process of equipping oneself with the necessary knowledge and skills to effectively navigate and succeed in the future. Consequently, it is crucial for universities to offer compulsory family education programs to their students, specifically focusing on assisting young males in gaining a deeper comprehension of their familial duties and goals. As college students transition into the marriage phase and become parents, their skills in family education will be beneficial for their future married life. By cultivating a positive mental foundation for the future, it significantly enhances the propensity of young women getting married.

Ultimately, government should prolong parental leave to mitigate the strain on women throughout the production period. Simultaneously, broaden the scope of community childcare services and enhance the caliber of vital public services, such as the accessibility of newborn and childcare facilities. The relevant government ministries should supervise firms in establishing an equitable promotion structure to prevent the marginalization of married women. Subsequently, the availability of social support may relieve women’s concerns about marriage.

## Data availability statement

The raw data supporting the conclusions of this article will be made available by the authors, without undue reservation.

## Author contributions

JC: Writing – review & editing, Writing – original draft.
